# Cholecystobronchocolic Fistula: A Late Complication of Biliary Sepsis

**DOI:** 10.1155/1994/39724

**Published:** 1994

**Authors:** D. A. Collie, D. N. Redhead, O. J. Garden

**Affiliations:** 1 Department of Radiology Royal Infirmary of Edinburgh Edinburgh Scotland; 2 Department of Surgery Royal Infirmary of Edinburgh Edinburgh Scotland; 3 Department of Radiology Royal Infirmary of Edinburgh Lauriston Place Edinburgh EH3 9YW Scotland

## Abstract

A case of a 48 year old woman presenting with bilioptysis due to a cholecystobronchocolic fistula is reported. Bilioptysis is a rare complication of biliary fistulae, with a high mortality due to chemical pneumonitis. Bronchospasm and rapid respiratory failure may ensue if aggressive management is not adopted. The site of fistulation is established by cholangiography, preferably by the percutaneous transhepatic route. Continued biliary drainage can lead to closure of these fistulae, or allow sufficient improvement in clinical condition to allow definitive surgery to be performed electively.


*HPB Surgery*, 1994, Vol. 7, pp. 319–326
Reprints available directly from the publisher
Photocopying permitted by license only

© 1994 Harwood Academic Publishers GmbH
Printed in the United States of America

### CASE REPORT

### CHOLECYSTOBRONCHOCOLIC FISTULA: A LATE COMPLICATION OF BILIARY SEPSIS

### Case Report of Diagnosis and Management

D.A. COLLIE*, D.N. REDHEAD* and O.J. GARDEN†
*Departments of *Radiology and †Surgery, Royal Infirmary of Edinburgh, Edinburgh*

*(Received 1 May 1993)*

A case of a 48 year old woman presenting with bilioptysis due to a cholecystobronchocolic fistula is reported. Bilioptysis is a rare complication of biliary fistulae, with a high mortality due to chemical pneumonitis. Bronchospasm and rapid respiratory failure may ensue if aggressive management is not adopted. The site of fistulation is established by cholangiography, preferably by the percutaneous transhepatic route. Continued biliary drainage can lead to closure of these fistulae, or allow sufficient improvement in clinical condition to allow definitive surgery to be performed electively.

KEY WORDS: Cholecystobronchial fistula, biliary fistula, complications

### INTRODUCTION

Bilioptysis is a rare symptom of biliary disease. The site of fistulation is usually between the intrahepatic biliary tree of the right lobe of liver and the bronchial tree of the right lung. Only one case of a cholecystobronchial fistula has been previously reported and this was in a patient with a hypoplastic right lobe of liver and suprahepatic gallbladder[1]. Prompt and accurate identification of the site of fistulation and decompression of the biliary tree is essential if life-threatening respiratory complications are to be avoided.

We present our experience of a patient with a cholecystobronchocolic fistula complicating abdominal sepsis and multiple abdominal operations.

### CASE REPORT

A 38 year old woman underwent, on two occasions, surgical drainage of an anterior abdominal abscess which extended from the right subdiaphragmatic space to the

Address correspondence to: Dr D.A. Collie, Department of Radiology, Royal Infirmary of Edinburgh, Lauriston Place, Edinburgh, EH3 9YW, Scotland pelvic brim. At neither operation was a source of sepsis established although on one occasion it was noted that the pus was bile-stained. There was no biochemical evidence of biliary obstruction or pancreatitis during either admission. The patient was lost to follow-up but represented to the same referring hospital four years later with gastric outlet obstruction. At a third laparotomy the anatomy of both the duodenum and gallbladder was obscured by a mass of dense fibrous tissue. Only the tip of the gallbladder could be identified and aspiration of this revealed normal appearing sterile bile. Further dissection proved technically impossible and vagotomy and gastrojejunostomy were performed for suspected duodenal ulcer disease.

Her post-operative progress was complicated by a persistently discharging wound sinus. Repeated sinograms demonstrated a large subphrenic cavity extending over the right lobe of the liver and which, on several occasions, was shown to communicate inferiorly with the duodenum. Communication with the biliary tree was not suspected. There was no biochemical evidence of pancreatitis and liver function tests remained normal both during her hospitalisation and on several occasions when checked at follow-up review. The patient refused further surgery. She tolerated the persistent purulent discharge from the sinus over a further two year period, but represented with expectoration of bile stained sputum. At this time a sinogram demonstrated a fistulous communication with the anterior basal bronchi of the right lower lobe but no communication with the biliary tree or duodenum was identified. Further surgical drainage of the abscess was undertaken but the fistula was not identified and again the dense mass of adhesions prevented safe dissection of the gallbladder.

Following this fourth operation the cutaneous discharge ceased but her bilioptysis increased to 200mls per day associated with the development of a right basal pneumonia and gradual deterioration of respiratory function. Abdominal CT demonstrated the inflammatory mass around the common bile duct, adjacent to and contiguous with the head of the pancreas (Figure 1). The body and tail of the pancreas appeared normal. Endoscopic retrograde cholangio-pancreatography was attempted but access to the duodenal papilla was prevented by distortion and stenosis of the pylorus.

As a result of her continuing deterioration and a total of ten years following her initial presentation she was referred to the Department of Surgery, Royal Infirmary, Edinburgh for further management.

In order to identify the fistula and decompress the biliary tree a percutaneous transhepatic cholangiogram (PTC) and insertion of an 8.3 F gauge Ring Catheter (William Cook Europe) were performed. A long stricture of the common bile duct was demonstrated, with partial filling of a contracted gallbladder containing multiple gallstones. The intrahepatic bile ducts were not dilated. A complex fistulous tract connecting the tip of the gallbladder to the transverse colon inferiorly, and the bronchial tree superiorly (Figure 2), and an additional biliogastric fistula extending from the distal left hepatic duct were identified. The patient suffered severe bouts of coughing and complained of a metallic taste during the procedure. Both sputum and biliary aspirate grew coliforms, enterococci and Bacteroides species.

In view of the patients generally poor clinical state, management was initially conservative. Percutaneous biliary drainage was continued, she received antibiotic therapy with ciprofloxacin (Ciproxin, Bayer), gentamicin (Cidomycin, Roussel) and metronidazole, physiotherapy and nutritional support by nasogastric tube

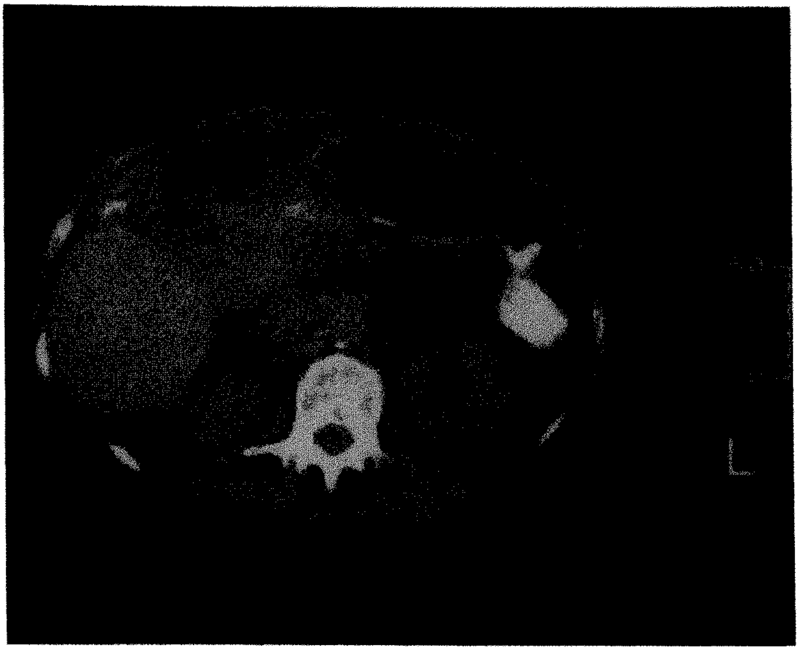

**Figure 1** Non-enhanced abdominal CT, showing a mixed attenuation mass surrounding the common bile duct, anterior to and continuous with the head of the pancreas, obscuring both the gallbladder and duodenum.

feeding. With this treatment she gained 6 kg in weight and the right basal consolidation cleared over a one month period. A repeat tubogram at this stage showed sealing of the biliobronchial communication but persistence of the biliogastric and cholecystocolic fistulae (Figure 3). Coeliac and superior mesenteric angiograms showed an aberrant right hepatic artery arising from the SMA, and in addition spleno-portal venous thrombosis with a plethora of collateral veins and associated splenomegaly.

Five weeks following initial decompression of the biliary tree by percutaneous transhepatic biliary drainage, laparotomy was undertaken. The fistula to the transverse colon was repaired, a cholecystectomy performed and drainage of the biliary tree effected by means of a hepaticojejunostomy-Roux-en-Y. Division of the biliogastric fistula was not possible due to dense adhesions between the stomach and left lobe of liver. Histology revealed chronic inflammatory changes only. A tubogram on the tenth post-operative day showed free flow of contrast into the jejunum with neither bronchial nor gastric fistulous communications and the percutaneous drain was removed. The post-operative period was complicated by a wound infection which settled with intravenous antibiotic therapy and the patient remains well and asymptomatic one year following discharge.

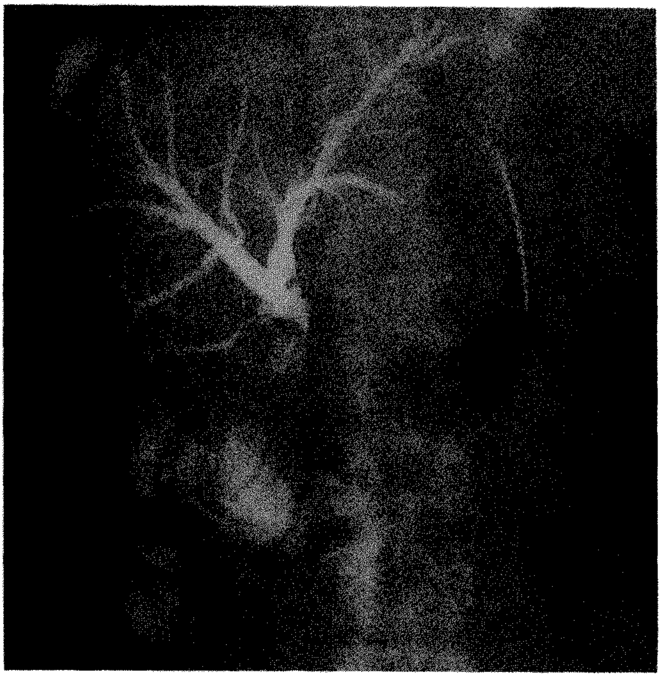

(a)

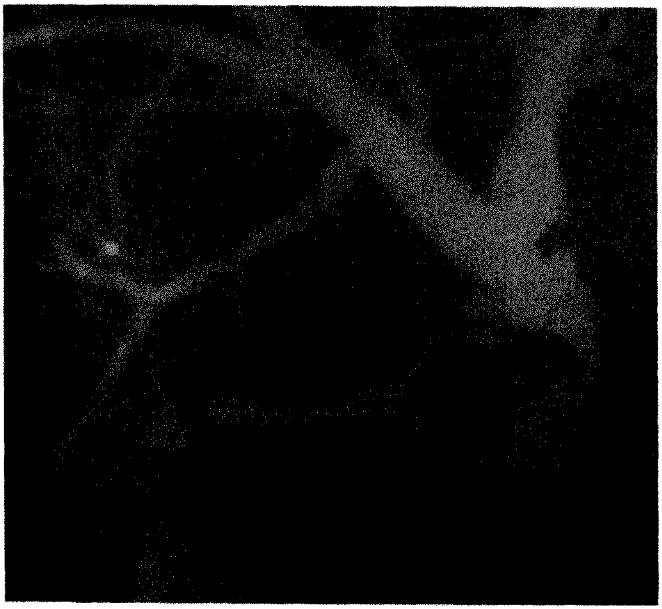

(b)

**Figure 2** Percutaneous transhepatic cholangiogram showing (a) a cholecystobronchocolic fistula, a small right subphrenic abscess, right basal consolidation and the biliogastric fistula. Bile duct catheter *in situ*. Detail of (b) cholecystocolic, (c) cholecystobronchial and (d) biliogastric communications.

(c)

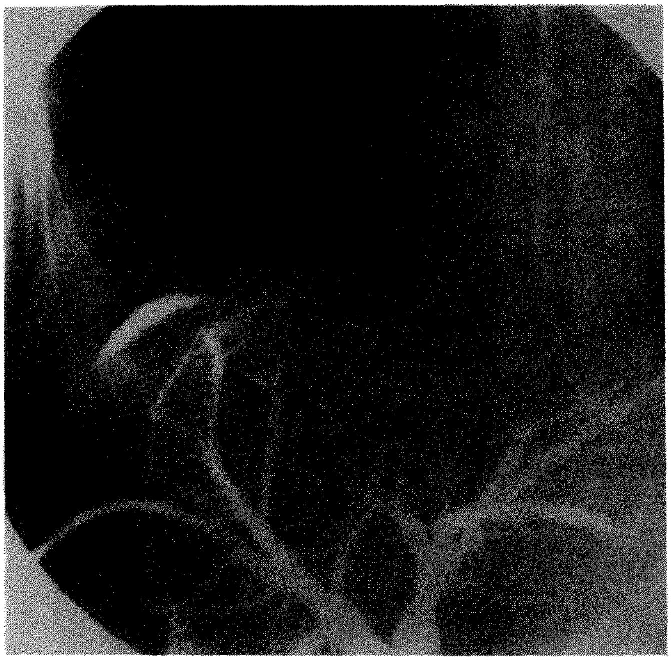

(d)

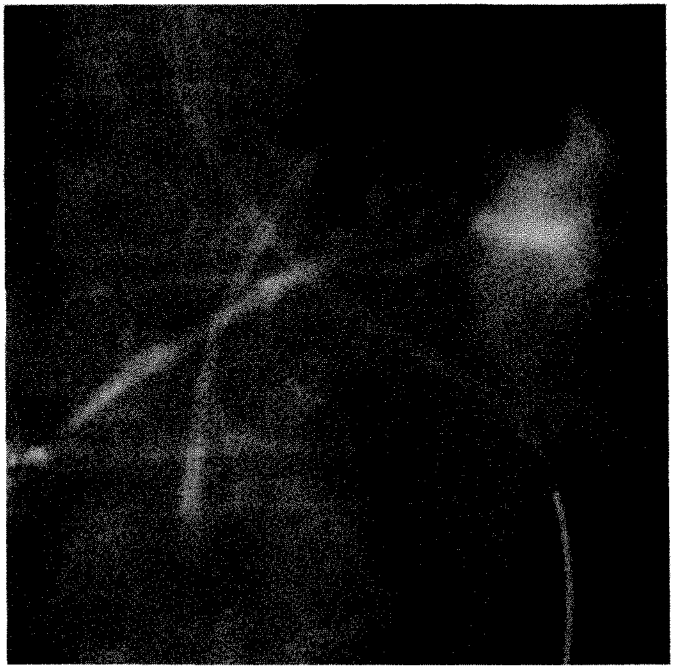

**Figure 2** (*continued*)

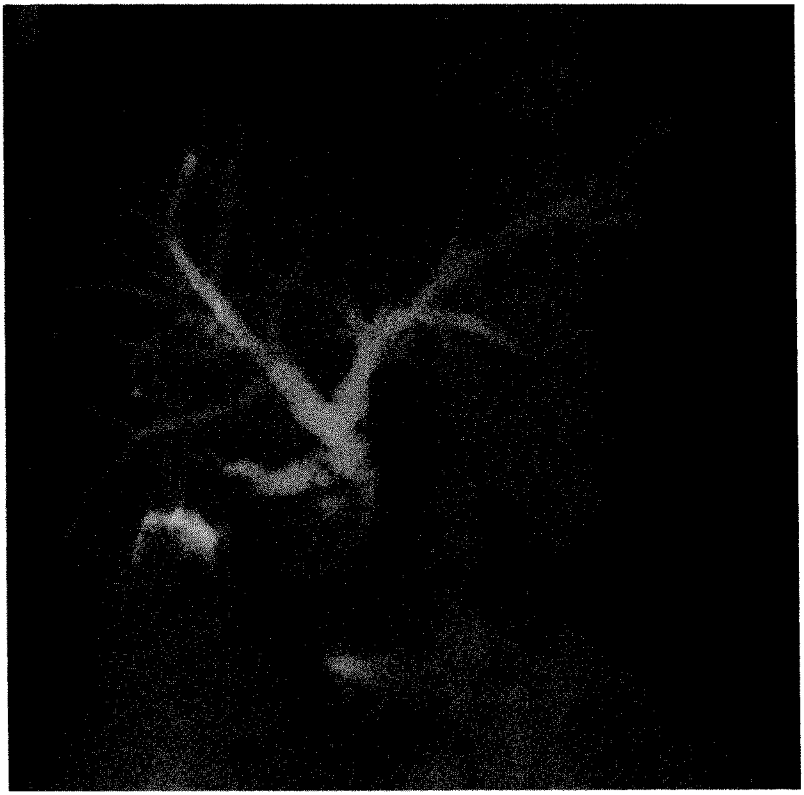

**Figure 3** Tubogram one month after continuous percutaneous biliary drainage. The cholecystobronchial communication has sealed but the cholecystocolic fistula persists.

### DISCUSSION

Biliary fistulae are diverse in both their aetiology and visceral communications. Infection is commonly implicated in their formation, usually with bacteria associated with cholangitis; E. coli, Klebsiella and enterococcal species.

Bronchobiliary fistulae have been reported due to fungal infections[2], tuberculosis[3], Entamoeba histolytica[4], and Echinococcus granulosa[5], the latter being the commonest cause of these particular fistulae worldwide. Occasionally malignant disease is the cause, particularly after chemotherapy[6]. Low choledochoduodenal fistulae are occasionally caused by duodenal ulceration[7]. Abdominal surgery has been contributary in a number of cases, and percutaneous biliary procedures are associated with biliopleural fistulas[8]. Trauma is the commonest cause of thoracobilia in the United Kingdom and North America[9].

Gallbladder fistulae represent a rare form of biliary fistula, and are almost universally associated with cholelithiasis and cholecystitis. Communication between the gallbladder and duodenum, stomach, colon or bronchus have been described, but a fistula joining the gallbladder, colon and bronchus concomitantly has never been previously reported.

The initial pathological process leading to this patient's biliary fistulae remains speculative, due, in part, to the paucity of clinical information from her first two admissions to the referring hospital. It is likely that stricturing of the common bile duct played an important early role, with cholelithiasis, cholecystitis (with empyema of the gallbladder) and chronic biliary sepsis instrumental in the late fistulation. However, the cause of the stricture remains obscure. Chronic pancreatitis can be associated with biliary strictures, but at no stage was there either CT or biochemical evidence of pancreatitis, and it is therefore unlikely that the pancreas was the source of sepsis in this case. It is concievable that a local duodenal perforation may have caused the common bile duct stricture but the subsequent course of events was not typical. Subsequent fistulation to the bronchial tree and to the stomach may have arisen from subphrenic abscess formation at some stage during the patients long illness. Portal vein thrombosis is likely to have arisen as a complication of the chronic sepsis. Whatever the aetiology, this case emphasises the importance of establishing a source of infection if an abscess is discovered at laparotomy either per-operatively or by subsequent imaging in the post-operative period.

In addition, the importance of adequate imaging in the work-up to complex biliary surgery, should be remembered. On two occasions cholecystectomy had proved technically impossible due to involvement of the extrahepatic biliary tree and portal vessels in the inflammatory mass. Coeliac and superior mesenteric angiography demonstrated the aberrant origin of the right hepatic artery and the plethora of collateral veins resulting from venous thrombosis. This latter finding had also been documented using colour flow doppler but as Tessler *et al.*[10] have recently emphasised, the absence of flow does not always indicate thrombosis and angiography should be performed for confirmation.

Identification of the site of fistulation has been described using several imaging modalities. Biliary scintigraphy has been advocated but the resolution is poor and cannot provide precise anatomical localisation of the site of leakage[11]. CT and ultrasound have been used in fistulae associated with echinococcal disease[5]. In the above case CT proved of benefit in identifying the extent of the inflammatory mass but not the fistulae. Ultrasound was technically difficult and may even have been misleading had imaging of the biliary tree been possible as this was undilated presumably due to decompression through the two fistulae. Cholangiography remains the definitive investigation of choice. Both endoscopic and percutaneous transhepatic approaches may be considered[12]. However, even if the endoscopic approach is successful, it is questionable whether it will provide the same detail as that obtained by the percutaneous transhepatic route; a large volume of contrast must be delivered rapidly in order to demonstrate such fistulous tracts. The final choice of approach obviously depends on local availability and expertise.

In addition to the diagnostic role of PTC, continued percutaneous drainage is an important means of allowing healing of fistulae in an obstructed biliary system[9]. Kaufman *et al.*[13] used this method in 12 patients, six of whom had subsequent spontaneous sealing of biliary leaks. In the remaining six patients the procedure allowed further surgery to be delayed until their condition improved sufficiently to allow definitive surgery to be performed. In the present case, it was unlikely that a non-operative approach to management would have proved successful in the long term. Dilatation and stenting of the biliary stricture[14] might have maintained the clinical improvement observed by biliary drainage alone. However, removal of the original focus of infection and surgical drainage of the biliary tree offered the best prospect of long-term cure once the patients condition had improved by judicious radiological intervention. We, like others[13], have been able to confirm the value of the presence of the biliary catheter in guiding dissection in difficult circumstances. However, the main value of percutaneous drainage in the case presented has undoubtedly been the recovery of what appeared to be an irretrievable situation.

### *References*

1. Allison, M.C., Milkins, S., Burroughs, A.K., Rogers, H.S. and Thomas, H.C. (1987) Bronchobiliary fistula due to acute cholecystitis in a suprahepatic gallbladder, *Postgraduate Journal of Medicine*, **63**, 291–294
2. Camarata, S.K., Dircks, J., Grambua, G. and Hyzy, R. (1991) Elevated right hemidiaphragm with yellow sputum production. *Chest*, **99**, 1463–1465
3. Tsunoda, T., Shiogama, T., Koga, M., Kohara, N., Eto, T., Motoshima, K. *et al.* (1991) Tuberculous liver abscess with bronchobiliary and gastrobiliary fistulae — a case report. *Japanese Journal of Surgery*, **21**, 100–104
4. Gugenheim, J., Ciardullo, M., Traynor, O. and Bismuth, H. (1988) Bronchobiliary fistulas in adults, *Annals of Surgery*, **207**, 90--94
5. Porta, E., Borgstrom, M.S., Giampoglia, F., Carillo, F. and Angalillo, M. (1982) Contribution of CT and ultrasound to the preoperative diagnosis of biliobronchial fistula caused by echinococcosis of the liver. *CT*, **5**, 349–350
6. Kocks, I., Korber, W., Larena-Avellanada, A. (1988) Bronchobiliary fistula in necrotizing liver metastasis of sigmoid cancer following regional chemotherapy. *Medizinische Klinic*, **83**, 499--501
7. Page, J.E., Dow, J., Dundas, D.D. (1989) Ulcerogenic choledochoduodenal fistula. *Clinical Radiology*, **40**, 58–60
8. Strange, C., Allen, M.L., Freedland, P.N., Cunningham, J. and Sahn, S.A. (1988) Biliopleural fistula as a complication of percutaneous biliary drainage: experimental evidence for pleural inflammation. *American Review of Respiratory Disease*, **137**, 959–961
9. Schwartz, M.L., Coyle, M.J., Aldrete, J.S. and Keller, F.S. (1988) Bronchobiliary fistula; complete percutaneous treatment with biliary drainage and stricture dilatation. *Radiology*, **168**, 751–752
10. Tessler, F.N., Gehring, B.J., Gomes, A.S., Perrela, R.R., Ragavendra, N., Bussutil, R.W. *et al.* (1991) Diagnosis of portal vein thrombosis; the value of colour flow doppler imaging. *American Journal of Roentgenology*, **157**, 293–297
11. Weissman, H.S., Chun, K.J., Frank, M., Koenigsberg, M., Milstein, D.M. and Freeman, L.M. (1979) Demonstration of traumatic bile leak with cholescintigraphy and ultrasonography. *American Journal of Roentgenology*, **133**, 843–847
12. Marks, W.M., Freeny, P.C., Ball, P.J. and Gannon, R.M. (1984) Endoscopic retrograde biliary drainage. *Radiology*, **152**, 357–360
13. Kaufman, S.L., Kadir, S., Mitchell, S.E., Chang, R., Kuinison, M.L., Camaron, J.L. *et al.* (1985) Percutaneous transhepatic biliary drainage for bile leaks and fistulas. *American Journal of Roentgenology*, **144**, 1055–1058
14. Bornman, P.C. (1989) Stenting in obstructive jaundice: ERCP verses PTC-no final answer. *HPB Surgery*, **1**, 167–170

